# Quantitative analysis of metastatic breast cancer in mice using deep learning on cryo-image data

**DOI:** 10.1038/s41598-021-96838-y

**Published:** 2021-09-01

**Authors:** Yiqiao Liu, Madhusudhana Gargesha, Mohammed Qutaish, Zhuxian Zhou, Peter Qiao, Zheng-Rong Lu, David L. Wilson

**Affiliations:** 1 Department of Biomedical Engineering, Case Western Reserve University, 10900 Euclid Avenue, Cleveland, OH 44106 USA; 2 BioInVision Inc, Suite E 781 Beta Drive, Cleveland, OH 44143 USA; 3 Department of Radiology, Case Western Reserve University, 10900 Euclid Avenue, Cleveland, OH 44106 USA

**Keywords:** Cancer imaging, Information technology

## Abstract

Cryo-imaging sections and images a whole mouse and provides ~ 120-GBytes of microscopic 3D color anatomy and fluorescence images, making fully manual analysis of metastases an onerous task. A convolutional neural network (CNN)-based metastases segmentation algorithm included three steps: candidate segmentation, candidate classification, and semi-automatic correction of the classification result. The candidate segmentation generated > 5000 candidates in each of the breast cancer-bearing mice. Random forest classifier with multi-scale CNN features and hand-crafted intensity and morphology features achieved 0.8645 ± 0.0858, 0.9738 ± 0.0074, and 0.9709 ± 0.0182 sensitivity, specificity, and area under the curve (AUC) of the receiver operating characteristic (ROC), with fourfold cross validation. Classification results guided manual correction by an expert with our in-house MATLAB software. Finally, 225, 148, 165, and 344 metastases were identified in the four cancer mice. With CNN-based segmentation, the human intervention time was reduced from > 12 to ~ 2 h. We demonstrated that 4T1 breast cancer metastases spread to the lung, liver, bone, and brain. Assessing the size and distribution of metastases proves the usefulness and robustness of cryo-imaging and our software for evaluating new cancer imaging and therapeutics technologies. Application of the method with only minor modification to a pancreatic metastatic cancer model demonstrated generalizability to other tumor models.

## Introduction

Cancer is the second leading cause of death in the US, and more than 90% of cancer deaths are due to metastases. Cancer cells moving into blood vessels or lymph vessels are capable of spreading to distant tissues, forming micro-metastases, potentially turning macroscopic after removal of the primary tumor^1^. Preclinical studies on metastases have elucidated various molecular mechanisms underlying cancer progression and metastasis^2,3^. Preclinical mouse models for metastatic breast cancer include tail vein injection, orthotopic, and intra-cardiac models^4^, which induce metastases at different locations. The tail vein injection model generally induces lung metastases^5^. The orthotopic model induces metastases in the lung, liver, and brain^6^. The intra-cardiac metastasis model produces bone metastases^7^. However, the current preclinical in vivo imaging modalities (e.g., magnetic resonance imaging [MRI], computed tomography [CT], positron emission tomography [PET], ultrasound, etc.) that are used to study cancer micro-metastases have limited resolution and contrast. Whole mouse histology is impractical, and image-guided biopsy is challenged by unknown accuracy/sampling issues. Tissue clearing combined with a light sheet microscope allows for 3D visualization of the fluorescence signal in thick tissues. However, most tissue clearing techniques involve complex processes that are time consuming and labor-intensive. The optimum clearing time for 1 mm-thick slices was identified as five days, which is the best compromise between the increase in light penetration depth due to lipid removal and a decrease in fluorescent signal as a consequence of protein loss^8^. Despite its limitations due to loss of fluorescence proteins, a recent immunolabeling technique vDISCO^9^ enhances fluorescence signals by adding a secondary antibody conjugated with bright fluorescent dyes. To clear and immunolabel a whole mouse using the vDISCO method, the blood heme must be further decolorized and the bones must be decalcified. The whole process could take up to two weeks and is, therefore, time consuming, technically difficult, and prone to failure. Moreover, it is impossible to register cleared fluorescent mouse images in in vivo modalities due to the elimination of anatomical information after the clearing process.

Cryo-imaging is a section-and-imaging technique that provides single cell resolution (as good as 5 µm), and a large field-of-view (up to a whole mouse view) in 3D color anatomy and fluorescence images^10^. Cryo-imaging has been utilized in multiple areas of cancer research, such as imaging agents^11–13^, imaging methods^14,15^, therapeutics^16–20^, and tumor models^21^. One of the major functionalities of cryo-imaging is to provide the ground-truth for the identification of metastatic tumors using fluorescent-protein-labeled cancer cells. However, high-resolution cryo-imaging of a whole mouse in color and fluorescence images could be as large as 120 GB and, therefore, manual analysis is time consuming. We are creating a Cancer Imaging and Therapy Analysis Platform (CITAP) that will allow researchers to study cancer biology and optimize pipelines of technologies (e.g., imaging agents, imaging methods, targeted nanotherapeutics, and tumor models), especially for metastatic and invasive cancers. Automatic segmentation of fluorescent-protein-labeled metastases is an important capability of CITAP software. CITAP substantially reduces the amount of human analysis time, provides significant insight, and makes repeated experiments easy and robust.

Machine learning-based tumor segmentation algorithms can be categorized into two types of methods that are suitable for isolated major tumors and for dispersive metastatic tumors. Major tumor segmentation is performed using voxel-wise classification, whereas dispersive metastatic tumors are segmented via a candidate detection and a false-positive reduction procedure. Voxel-wise classification schemes include hand-crafted feature extraction with machine learning classifiers^22^ and deep learning^23^. Deep learning is gaining momentum because no hand-crafted features are required to achieve state-of-art accuracy. In deep learning, fully convolutional neural networks (FCN)-based methods have been applied in various major tumor segmentation tasks, such as breast tumors in mammograms^24^, brain tumors in MRI^25^, hepatic tumors in CT^26^, and pancreatic tumors in CT^27^. The CT lung nodule is a representative example of dispersive tumors. Conventional approaches for detecting candidates include the Hounsfield unit (HU) value thresholding-based method^28^ and shape-based method^29^. Deep learning approaches for candidate detection incorporate a segmentation network, such as U-Net^30^ or a detection network such as faster regional-CNN^31^. At the false-positive reduction stage, due to the 3D nature of the CT-imaging modality, 2D- and 3D-CNNs have been studied and compared. For 2D CNNs, spatial information is lost with 2D single slice input. However, due to many existing pre-trained models, researchers have tried to include more spatial information through inputting data from the adjacent three slices and implementing tri-planar schemes. Three-dimensional networks capture the volumetric information but are computationally more expensive.

Segmentation of fluorescent metastases in a whole mouse body is a unique problem and there are not many relevant publications. To segment dispersive fluorescent stem cells in whole mouse cryo-imaging, Patiwet Wuttisarnwattana et al*.*^32^ employed an algorithm for detection of candidate stem cells using sombrero filtering and top-hat transform, followed by bagging decision tree classification of candidates. Chenchen Pan et al*.*^33^ recently utilized deep learning to segment fluorescent breast cancer metastases in a whole mouse body with light sheet microscopy and tissue clearing. They chunked the large dataset into sub-volumes of 350 × 350 × 350 voxels at 10 × 10 × 10 µm resolution and used three 2D U-Net-like neural networks to segment the maximum intensity projection images along three axes. After tissue clearing and the vDISCO method to enhance the fluorescence signal of cancer cells^9^, there was little confounding auto-fluorescence, which allowed their maximum intensity projection method to work. In our case, high-resolution cryo-imaging with 10 × 10 µm in-plane resolution could image single metastatic cells, and co-registered anatomical color images were automatically acquired along with fluorescence images. Although auto-fluorescence in cryo-imaging can be confounding, expert human readers who are trained in auto-fluorescence signals from a healthy control mouse can distinguish auto-fluorescence and cancer fluorescence by examining the 3D shape of the signal.

Previously, we utilized high-resolution, sensitive cryo-imaging, developed whole mouse non-rigid registration algorithms between in vivo MRI and cryo-images^34^, and demonstrated the performance of CITAP in validating a molecular contrast agent, CREKA, in MR (CREKA-Gd) and fluorescence (CREKA-Cy5) imaging of breast cancer metastases^13^. Our previous work required days of manual analysis to segment the metastatic tumors, which precludes its use in high-volume studies. Here we develop and employ a deep learning-based, highly automated segmentation of metastases to evaluate and quantify the distribution of metastases in a whole mouse body from breast cancer intra-cardiac tumor model. Further, to evaluate generalizability of the method, we will test the method on a different mouse model (i.e., intrahepatic KPC-GFP pancreatic metastasis).

## Metastases segmentation and classification algorithm

The segmentation of green fluorescent protein (GFP)-labeled metastases from fluorescence whole mouse volume involves multiple steps: (1) exclude exterior fluorescent regions (cryo-gel, skin, and fur); (2) segment big-metastases candidates with marker-controlled 3D watershed algorithm; (3) segment small-metastases candidates with multi-scale Laplacian of Gaussian (LoG) filtering followed by Otsu segmentation; (4) classify big- and small-metastases candidates using 3D CNN-based methods; and (5) perform computer-assisted corrections. Due to the high-resolution of cryo-image data in a whole mouse, image volumes can be as large as 120 GB of color and fluorescence image data. Full resolution is needed to capture small-metastases. However, the size of RAM poses limitations and therefore in some steps, we resort to processing chunks of full resolution data at a time. In other steps (i.e., steps 1 and 2), we perform processing of down-sampled color and fluorescence volumes at 40 × 40 × 50 µm resolution. When processing small-metastases (steps 3 and 4), we process data chunks at full resolution (10 × 10 × 50 µm). In this section, we describe the algorithms. Cryo-imaging and computational experiments are described in the following sections.

### Exclude immaterial, exterior fluorescent regions

We use 3D color and green fluorescence channel (GF) images to mask out the exterior embedding cryo-gel, skin, and fur. An intensity threshold of > 110 is applied to the green channel of the color images, where the contrast between mouse body and the cryo-gel is greatest. In addition to identifying the white cryo-gel, some bright parts in the mouse body, such as bone, are also segmented. To exclude these bright body parts, a 3D connected component operation is applied to the binary segmentation. The cryo-gel components are always the largest components, and other components are from the mouse body that should be excluded. Skin and fur are masked by morphologically dilating the cryo-gel mask with a disk-shaped structuring element with a radius of 2 voxels (80 µm). The output is a binary mask volume with immaterial regions labeled as zero and the mouse body labeled as 1.

### Segment big-metastases candidates

After masking out the immaterial exterior fluorescent regions, we apply a method to segment big-metastases that tend to be very bright. In the following steps, we apply marker-controlled 3D watershed segmentation to the GF volume. (1) We smooth the GF volume using a 3D Gaussian filter with a sigma of 160 µm. (2) We apply an empirically determined intensity threshold of 20 to the GF volume to capture the brightest voxels in large, bright tumors. Morphological hole filling (a 3D structuring element of 400 µm diameter) and 3D connected components are sequentially performed on the thresholded volume. Connected components with volume < 0.8 mm^3^ are excluded, as they do not correspond to big metastases. The remaining connected components are morphologically eroded (3D structuring element of 120 µm diameter) and set as foreground markers. (4) We create a gradient magnitude volume by filtering the Gaussian smoothed combined volume with a 3D Prewitt operator (3 × 3 × 3 voxels). (5) We modify the gradient magnitude volume to have regional minima in the foreground and background marker regions (the background marker is the volume border). (6) We run marker-controlled 3D watershed with 26 neighbor connectivity using the modified gradient magnitude volume as input and obtain individual segmented tumors in a labeled volume. (7) We apply rules to merge the over-segmented watershed results. In other words, we identify background watershed fragments that are in the immaterial cryo-gel region and inside the mouse body with a volume > 10% of the mouse body. These voxels are assigned to a background label of 0 to exclude them from the following merging process. Each of the remaining watershed fragments are dilated by 0.12 mm, and all fragments in the dilated neighborhood are merged into one component. The output is a binary volume with the big-metastases candidate labeled as 1 and the background labeled as 0. The size of the Gaussian filter and morphological structuring element are empirically determined to obtain the best watershed result.

### Segment small-metastases candidates

Small-metastases candidates are detected and segmented in full-resolution, chunked cryo-imaging GF volumes. Steps include (1) preprocessing, (2) detection and segmentation in chunks, (3) combining segmentation results across chunks, and (4) post-processing.

The preprocessing step consists of three phases. First, we generate the volume of interest for segmentation by masking out the exterior fluorescent regions and the big-metastases candidates (after up-sampling). We then chunk the full resolution volume into smaller data to fit into RAM. Each data chunk contains a stack of slices with overlap between any neighboring two chunks. The calculation process for the number of chunks and the number of slices in each chunk of our experiment are described in Supplementary Material.

Second, small-metastases candidates are detected and segmented within each chunk. Detection is performed using 2D multi-scale LoG filters with σ = 2, 4, 6, 8, 10 to account for various sizes of small metastases. For each voxel, the maximum filter response is selected across five σ scales. Threshold T_1_ is applied to generate candidate seeding points. To segment the candidates, we dilate the candidate seeding points with a 3D structuring element and then apply Otsu segmentation on the dilated neighborhood. The 3D structuring element is roughly spheroidal with a height of five slices and radii of 3, 7, 12, 7, and 3 voxels in each slice. The segmentation yields binary volumes, with candidates labeled as 1 and the background labeled as 0.

Third, after reclaiming the used memory from the intermediate results, which are shown in Table S1, we combine the segmentation results from all chunks by performing a logical OR operation to merge segmentations of overlapping slices in adjacent chunks.

Fourth, for post-processing, we remove candidates connected to exterior fluorescence or big-metastases candidate labels. We perform morphological closing on the results of the previous step with a 3D structuring element with size 40 µm × 40 µm × 200 µm to account for the inhomogeneous GF signals inside some candidates. We remove any candidates with a volume < 4 × 10^6^ µm^3^, thus giving an effective diameter of 98 µm assuming a sphere, because the smallest detectable tumor is 4 × 10^6^ µm^3^ due to a low fluorescence signal.

Combined with the up-sampled big-metastases candidate volume, the final candidate segmentation result is a full-size, full resolution volume with the big- and small-metastases candidates labeled as 1 and the background labeled as 0, which would be approximately 1 GB. Three-dimensional connected component operation is performed on the final candidate segmentation result to generate distinct labels for each component.

### Classify the big- and small-metastases candidates using CNN-based methods

**Figure 1:**
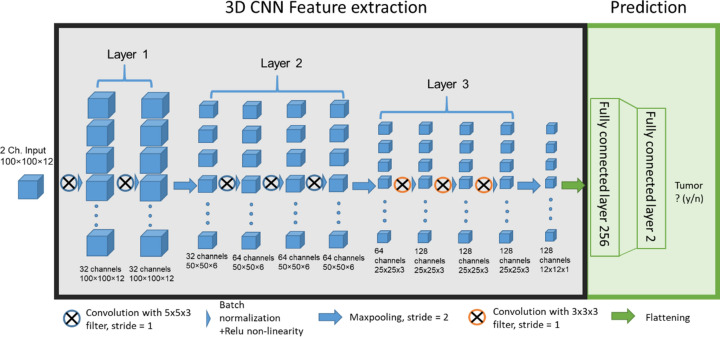
The CNN architecture for classification of a candidate volume as “tumor” or “not tumor”. The CNN is decomposed into two parts: 3D CNN feature extraction and prediction.

**Figure 2:**
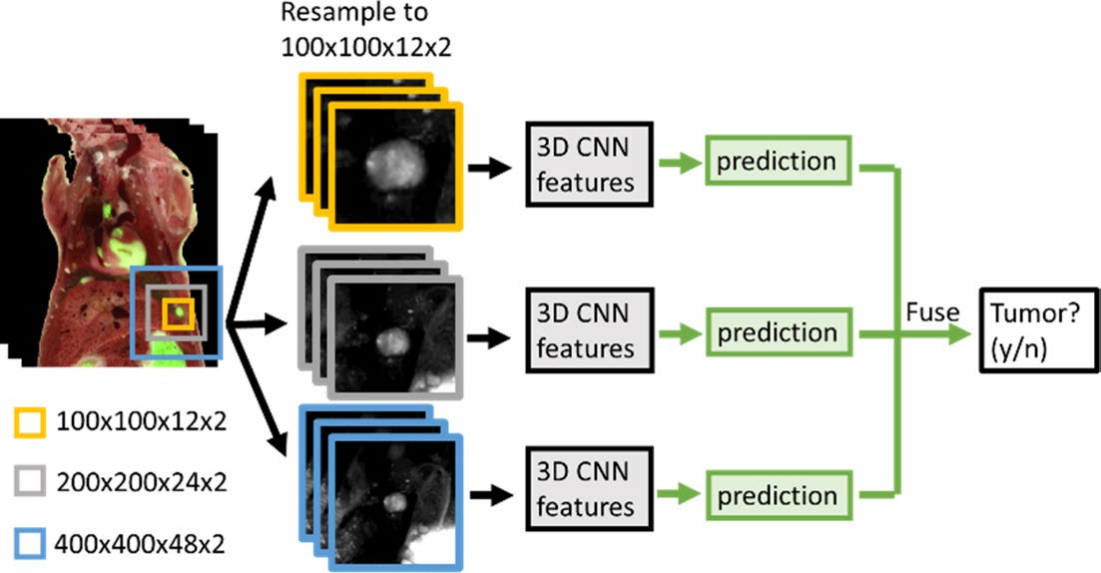
The workflow for 3D multi-scale CNN. The inputs are multi-scale 3D volumes around each candidate resampled to 100 × 100 × 12 with 2 channels – color and GF. The resolution for the resampled inputs at three scales are 10 µm × 10 µm × 50 µm, 20 µm × 20 µm × 100 µm, 40 µm × 40 µm × 200 µm, respectively. The predicted probabilities from three scales are fused using Eq. (1) to generate final probability.

**Figure 3:**
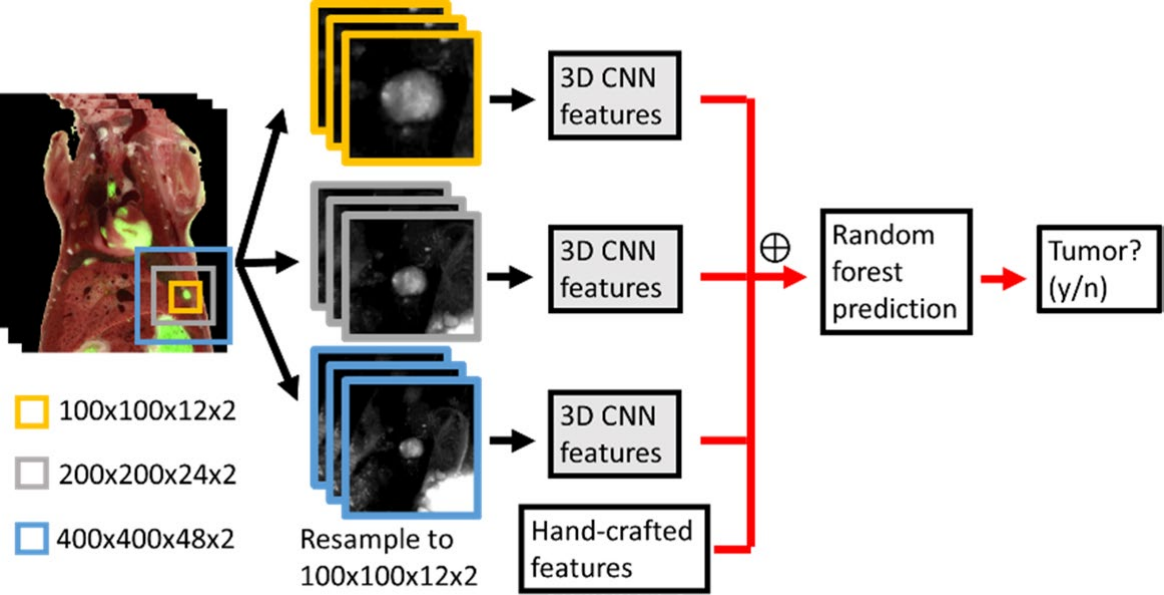
The workflow for random forest classifier with multi-scale CNN features + hand-crafted features. Additional hand-crafted features are combined with 3D CNN features for random forest classifier prediction.

For each candidate, we extract the surrounding color and GF volumes for CNN processing. We compare two algorithms: (1) 3D multi-scale CNN, and (2) 3D multi-scale CNN features + hand-crafted features with a random forest classifier. The basic component for both algorithms is the 3D CNN. For each candidate, a 3D neighborhood around the center of mass of the candidate is extracted for the classification step. We use the CNN architecture shown in Fig. 1. The CNN is decomposed into two parts: 3D CNN feature extraction and prediction. This decomposition allows for a better demonstration of the difference between our two algorithms, as shown in Figs. 2 and 3. In Fig. 2, to encode multi-scale contextual information from candidates of various sizes, we use three neighborhood sizes to train three CNNs separately and fuse the predicted probabilities for final predictions, as shown in Fig. 2. The three neighborhood sizes are 100 × 100 × 12 voxels, 200 × 200 × 24, and 400 × 400 × 48, respectively, and all inputs are resampled to 100 × 100 × 12 voxels for input into the CNNs. The CNN structure used for the three scales is the same. The receptive field size for our 3D CNN is 64 × 64 × 48 and was deemed reasonable for our input with a size 100 × 100 × 12. The input 3D volume for each candidate contains two channels, GF and grayscale anatomy. The grayscale anatomy image is computed using (Gray = 0.2126∙R + 0.7152∙G + 0.0722∙B)^34^. Both channels are divided by 255 to normalize the intensity range to 0–1. If the boundaries of the neighborhood volume exceed the boundaries of the cryo-images, voxels outside the cryo-images are assigned to an intensity of zero. To fuse the prediction from three scales, we adopt the method in^35^ with weighted average of the predicted probabilities for each candidate, which are calculated as follows:

1 $${p}_{fuse}=0.3\times {p}_{1}+0.4\times {p}_{2}+0.3\times {p}_{3}$$

where *p*_*fuse*_ is the fused probability; *p*_*1*_ is predicted probability from scale 100 × 100 × 12; *p*_*2*_ is the predicted probability from scale 200 × 200 × 24; *p*_*3*_: predicted probability from scale 400 × 400 × 48.

Figure 3 shows the workflow for the algorithm “3D CNN features + hand-crafted features” using the random forest classifier. CNN features from the three scales are extracted after training the three CNNs. Hand-crafted features include location, intensity, and morphology features. For location, we compute the normalized (x, y, z) coordinates of the center of mass for each candidate by dividing the spatial (x, y, z) coordinates with the corresponding (x, y, z) dimensions of the whole mouse. To extract intensity features, we calculate the min, max, mean, and standard deviation in red and green channels in color images and in the GF images. For morphology features, we calculate volume, principal axes length, surface area, orientation represented by Euler angles, extent (ratio of number of voxels in the region to number of voxels in the bounding box volume), and solidity (proportion of number of voxels in the convex hull that are also in the region). We create an additional feature—mean GF intensity per voxel, which combines the GF intensity and candidate volume to account for the fact that big metastases are brighter than small metastases, which are calculated using Eq. (2). There are altogether 256 × 3 = 768 CNN features and 29 hand-crafted features.

2 $$Mean\, GF\, per\, voxel= \frac{Mean\, GF\, intensity }{Volume}$$

Ground-truth labeling of a candidate as cancer (+) is determined by two rules: The center of mass of the candidate must be within 60 µm of the center of mass of a manually annotated tumor OR if the modified IoU is > 0.5. The modified IoU is calculated as intersection/min (candidate volume, manual annotation volume). These two rules are proposed to deal with over-segmented candidates in heterogeneous metastases. Manual annotation of metastases was performed by Yiqiao, an expert in reading cryo-images, who examined the GF signals in cancer mice and confirmed their presence through the absence of GF signals in the same anatomical location of the healthy mouse. Of course, no candidates from the healthy mouse arise from GFP-labeled tumors. Hence, they are all deemed as cancer (−).

To deal with class imbalance where the number of cancer (−) candidates is much greater than the number of cancer (+) candidates when training the CNN, we employ weighted cross entropy loss and on-the-fly positive candidate random oversampling to augment the data. The weighted cross entropy loss function is shown in Eq. (3). Class 1 represents cancer (−), and class 2 represents cancer (+). Image augmentations are 2D-based and types include zooming, rotation, horizontal and vertical flipping, and brightness scaling. The ranges for zooming, rotation, and brightness scaling are 0.9–1.1, − 90° to 90°, and 0.8–1.2, respectively. Three-dimensional augmentation of a candidate volume is performed by randomly selecting a set of augmentation parameters and applying them to all 3D slices.

3 $$cross \,entropy\, loss=-{\sum }_{i=1}^{k}{w}_{i}{Y}_{i}\mathrm{log}\left({P}_{i}\right)=-[{w}_{1}{Y}_{1}\mathrm{log}\left({P}_{1}\right)+{w}_{2}{Y}_{2}\mathrm{log}\left({P}_{2}\right)]$$

where w_1_ and w_2_ are the weights for classes 1 and 2, respectively; Y_1_ and Y_2_ are the ground-truth labels for classes 1 and 2, respectively; P_1_ and P_2_ are the predicted probabilities for classes 1 and 2, respectively.

## Experimental methods

The mouse experiments for GFP-labeled breast cancer metastases is detailed in Zhuxian et al^13^. Briefly, we injected 1 × 10^5^ 4T1-GFP-Luc2 cells into the left ventricle for breast cancer metastases. The tumor growth was monitored with bioluminescence (BLI) using a Xenogen IVIS Lumina system. The mice with BLI confirmed breast cancer metastases were sacrificed after 2–3 weeks. The mice were then embedded in the optimal cutting temperature cryo-gel, and flash frozen with liquid nitrogen for cryo-imaging. All the mice were obtained from Charles River and housed in the Animal Core Facility at Case Western Reserve University.

For the pancreatic cancer metastasis model, we injected 2 × 10^4^ KPC-GFP-Luc cells into the portal vein of outbred athymic nude mice (The Jackson Laboratory, Bar Harbor, ME). Specifically, a ventral laparotomy was performed with a scalpel to visualize the liver and portal vein. A 2 × 10^6^ KPC-GFP-Luc cell/mL suspension in ice-cold phosphate buffered saline was mixed with Evans Blue dye at a 1:10,000 dilution. A micro-syringe was used to inject 10 µL of the cell suspension directly into the portal vein. The needle was held in place for 3 s and then withdrawn. The mouse was sacrificed for cryo-imaging after 1 month.

All animal experiments were performed in accordance with the animal protocol approved by the CWRU Institutional Animal Care and Use Committee. The study was carried out in compliance with the ARRIVE guidelines. All experiments were performed in accordance with relevant guidelines and regulations.

For cryo-imaging, the frozen mice were sectioned and imaged at 10.472 × 10.472 μm in-plane resolution and 50-μm section thickness using the CryoViz™ (Bioinvision Inc, Cleveland, OH). Color, green, and red fluorescence (RF) images were acquired using a liquid–crystal RGB filter and monochrome camera. Fluorescence images of excitation and emission were acquired using dual band FITC/Cy5 fluorescence filters. The GF and RF volumes capture emissions in wavelength 500–570 nm and 670–780 nm, respectively. In this experiment, RF was reserved for the CREKA-Cy5 contrast agent, and therefore we only used GF for metastases segmentation.

Calibrations of the intensity in color images and GF images were performed with cryo-gel as the reference. Cryo-gel makes up the ivory background in the color cryo-images. We set the standard GF intensity of the cryo-gel as 5, color red/green/blue channels = 150/120/120. A region of pure cryo-gel from the mouse to be processed was selected manually. The mean intensity of GF and color red/green/blue channels of the cryo-gel were compared to the standard intensity and calibrated accordingly.

## Computational methods and experiments

We used validation data to optimize hyperparameters in 3D CNN prediction and random forest prediction. For 3D CNN prediction, we optimized the batch size, learning rate, and positive class weights. Though we used multi-scale CNN, the hyperparameters across the three scales were kept the same. Batch sizes of 8 and 16 were compared, and learning rates of 10^−4^, 10^−5^, and 10^−6^ were compared, and positive class weights varied in the range of 5 to 30 with a step size of 5. For random forest prediction, we optimized the number of decision trees, max depth of a decision tree, max number of features at each split, min number of samples required at leaf node, min number of samples required to split an internal node, and positive class weights in the ranges of 50–1000, 1–100, 1–500, 5–150, 5–150, and 10–1000, respectively. A “tree of parzen estimators” algorithm in hyperopt^36^ with 100 iterations were utilized for the optimization of the random forest. To optimize the hyperparameters in both 3D CNN and random forest, the area under curve (AUC) from receiver operating characteristic (ROC) plot was used as the objective function.

Training/validation/test were split in four breast cancer-bearing mice as 2/1/1. A healthy control mouse (i.e. no tumor, no GFP labeling) was also used for testing. We compared the performance with and without color anatomy channel as input in 3D CNN prediction and random forest prediction by evaluating the AUC of ROC plot, AUC of precision-recall curve, and the number of false positive (FP) candidates at given sensitivity on one test mouse. After identification of the optimal method, the performance of classification was evaluated using fourfold cross validation. In each fold, the four cancer mice were used as test data, one at a time. The mouse with the smallest number of cancer (+) candidates compared to that of the other three mice was used as validation data to optimize the hyperparameters in each fold. To save on the computational cost of hyperparameter optimization in 3D CNN, we fixed the batch size and learning rate to previously optimized results, and only the class weights varied in the range of 5 to 30, with a step size of 5. We reported the sensitivity, specificity, F1 score, and AUC. We developed a MATLAB graphical user interface (GUI) for easy and fast semi-automatic exclusion of FP candidates and used Amira software to include false negative (FN) candidates at the same time.

We built our CNN network with Keras Tensorflow. Adam optimizer was employed with the following stopping criteria for training—loss of validation did not increase in 10 epochs or 100 epochs was reached, whichever comes first. The nominal number of epochs used was around 60. The number of metastases candidates in the four breast cancer mice are shown in Table 1. For training and testing the CNN, we used the NVIDIA Geforce RTX graphics card with 12 GB memory.

**Table 1:** The number of big-metastases candidates, big + small metastases candidates, ground-truth cancer (+), and ground-truth cancer (−) candidates in four cancer-bearing mice and one healthy mouse.

	Cancer mouse1	Cancer mouse2	Cancer mouse3	Cancer mouse4	Healthy mouse
# Big-metastases candidate	45	29	42	33	21
# Big + small-metastases candidate	8337	6576	5270	5139	10,264
# Ground-truth cancer (+) candidate	202	125	121	239	0
# Ground-truth cancer (−) candidate	8135	6459	5149	4900	10,264

## Results

**Figure 4:**
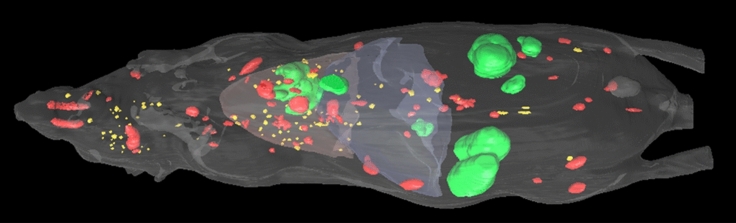
3D visualization of manually annotated metastases in one mouse. Tumor labels are rendered in green (> 2 mm), red (0.5–2 mm), and yellow (< 0.5 mm). The brown and blue semi-transparent regions represent lung and liver, respectively.

Manually annotated tumor labels are rendered in green (> 2 mm), red (0.5–2 mm), and yellow (< 0.5 mm), as shown in Fig. 4. The red and blue semi-transparent regions represent the lung and liver, respectively. The total number of manually identified metastases was 239, and were distributed in the lung, liver, lymph node, adrenal gland, bone, and brain. In the following sections, we analyze the performance of candidate segmentation and classification, and quantify the size and distribution of metastases.

### Metastases candidate segmentation

**Figure 5:**
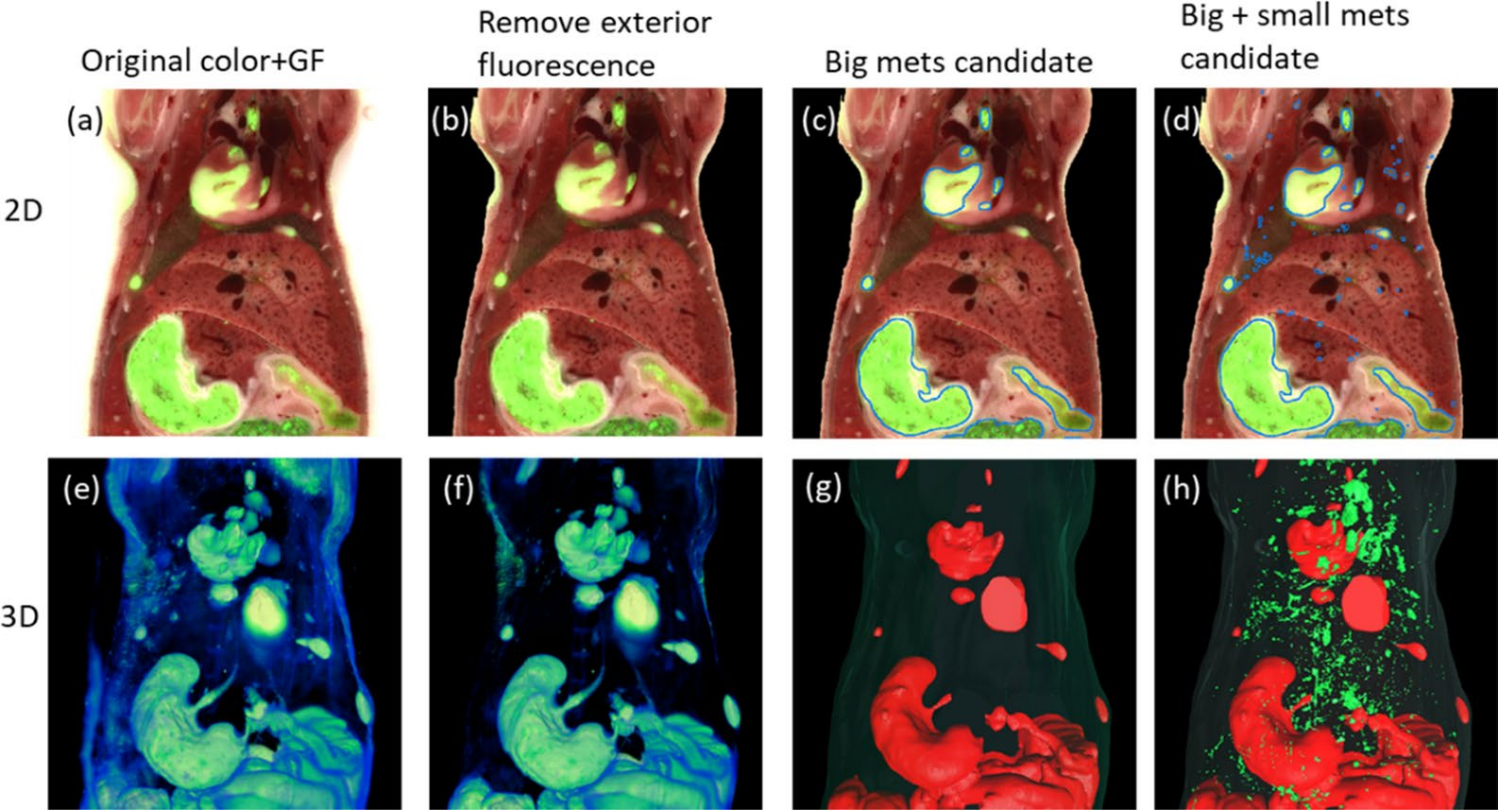
Workflow and results of metastases candidates segmentation. The first and second row shows 2D images and 3D rendering, respectively, in the abdominal region. The fused color and GF image and the 3D volume rendering of GF image are shown in (**a**) and (**e**), respectively. 2D and 3D results after exclusion of exterior fluorescence are shown in (**b**) and (**f**), respectively. 2D contouring and 3D surface rendering of the watershed segmented big metastases candidates are shown in (**c**) and (**g**), respectively. 2D contouring and 3D surface rendering of segmentation of small metastases candidates are shown in (**d**) and (**h**), respectively. There are a lot of FPs from auto-fluorescent structures such as spine, bile duct, lung vessels and airways etc. The segmentation results are input to the CNN classification algorithm.

Table 1 shows the numbers of candidate and actual metastases in the data. Among the 4 cancer-bearing mice and healthy mouse, the number of segmented big-metastases candidates and big + small-metastases candidates are shown in the first two rows of Table 1. Visualization of the intermediate results of cancer mouse 4 is shown in Fig. 5. The original 2D fusion of color anatomy and GF images and 3D volume rendering of the GF images are shown in the first column. Exclusion of the exterior fluorescent regions, the segmentation results of big-metastases candidates and big + small metastases candidates are shown in the following columns. The first row illustrates 2D fused color + GF images along with candidates in blue contour; the second row illustrates 3D volume renderings of GF images and surface rendering of the body label and metastases candidate label.

**Figure 6:**
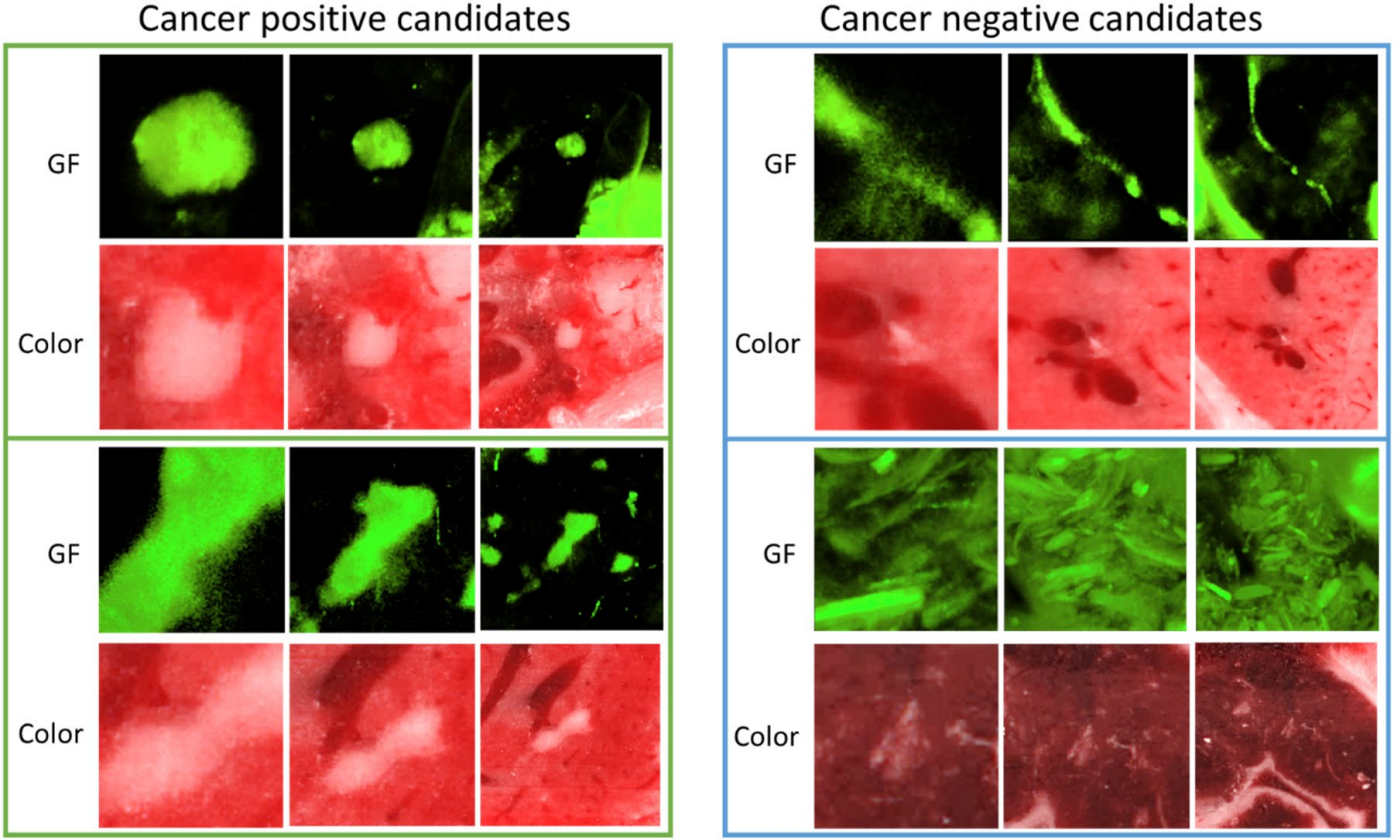
Representative cancer positive and negative candidates. The input from three scales having different volumes of view are presented in three columns with left to right as 100 × 100 × 12, 200 × 200 × 24, and 400 × 400 × 48. GF images are volume rendered visualizations, and color anatomy images are the central 2D slice from the 3D volume. The two positive candidates are from lung and liver, respectively. The two negative candidates are from bile duct in liver and food remnant in GI tract, respectively.

After comparing with the manual annotation, the number of ground-truth cancer (+) and ground-truth cancer (−) candidates are shown in the last two rows in Table 1. Visualization of some representative cancer (+) and cancer (−) candidates are shown in Fig. 6. The 3D volume rendering of multi-scale GF images and 2D central slices of the multi-scale color images from two cancer (+) and (−) candidates are shown. Two cancer (+) candidates are from the lung and liver, respectively, which have strong GF signals and white color signals. Negative candidates originate in auto-fluorescent bones, bile ducts in the liver, gallbladder, alfalfa-free food remnants in the GI tract, lung airways, and ear. The two cancer (−) candidates are from bile duct in liver and alfalfa-free food remnant in the GI tract.

### Metastases candidate classification

**Figure 7:**
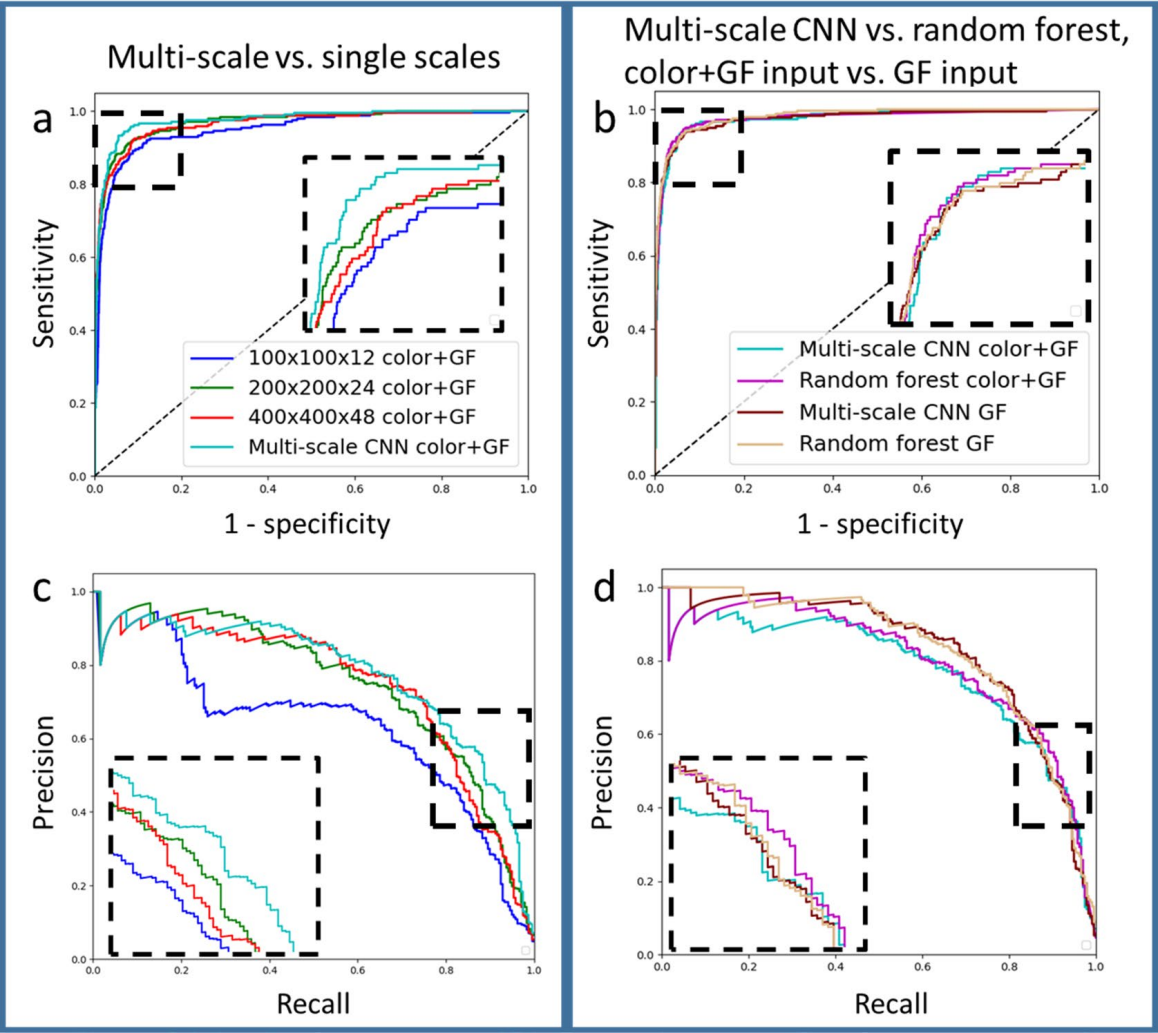
ROC and PRC show the effect of CNN size, color image as input, and random forest classifier configurations. ROC’s and PRC’s comparing CNNs with 100 × 100 × 12, 200 × 200 × 24, 400 × 400 × 48, and multi-scale color + GF input are shown in (**a**) and (**c**), respectively. Improvement with multi-scale is evident. ROC’s and PRC’s comparing multi-scale CNN vs. random forest and color + GF input vs. GF input are shown in (**b**) and (**d**), respectively. Random forest classifier with color + GF input is the best in the zoomed region. AUC values of ROC and PRC are shown in Table 2. The legends are the same between (**a**) and (**c**), (**b**) and (**d**).

Our three strategies for improving classification performance include (1) multi-scale CNN, (2) color + GF image input, and (3) random forest (short for random forest classifier with multi-scale CNN features + hand-crafted features). We used cancer mice 1 and 2 for training, 3 for validation, and 4 for testing to demonstrate this result. The optimized CNN hyperparameters were batch size 8, learning rate 10^−5^, and positive class weight 20. The optimized random forest hyperparameters included the number of decision trees (752), max depth of a decision tree (50), max number of features at each split (4), min number of samples required at leaf node (32), min number of samples required to split an internal node (135), and positive class weight (50). The AUCs of ROC and precision-recall curves of the single-scale CNNs, multi-scale CNN, color + GF image input, GF image input, and random forest are shown in Table 2. We also show some of the ROC curves and precision-recall curves (PRC) in Fig. 7. Using the color + GF image as input, the ROC curves and PRC for CNNs with input sizes 100 × 100 × 12, 200 × 200 × 24, 400 × 400 × 48, and multi-scale are shown in Fig. 7A and C, respectively. The ROC curves and PRC for comparing color + GF vs. GF as input and comparing multi-scale CNN vs. random forest are shown in Fig. 7B and 7D, respectively. The multi-scale CNN performed better than the single-scale CNNs. Out of the three scales, 200 × 200 × 24 were the best. Although the GF input has better AUCs than color + GF input, in the region where sensitivity/recall is in the range of interest 0.85–0.95, the color + GF achieved better performance, as shown in the zoomed-in black areas in Fig. 7B, D. We observed that the random forest performed better than the multi-scale CNN. Next, we demonstrate the number of FPs at different sensitivities for color + GF and GF input, and multi-scale CNN and random forest in Table 3. At a sensitivity level of 0.85–0.95 (which is of most interest), random forest with color + GF input achieved the lowest number of FPs. Since correcting for FN candidates requires searching the whole mouse, correcting for FP candidates is easier than correcting for FN candidates. We selected the probability threshold corresponding to sensitivity 0.9, which resulted in 23 FN and 178 FP candidates. The number of FP candidates in the healthy control mouse was 205 based on the threshold using the same probability.

**Table 2:** Effect of CNN size, color image as input, and random forest with hand-crafted features on classification performance.

	100 × 100 × 12	200 × 200 × 24	400 × 400 × 48	Multi-scale CNN	Random forest with multi-scale CNN + hand-crafted features
**Color + GF input**					
AUC ROC	0.9499	0.9684	0.9655	0.9765	0.9754
AUC precision-recall	0.6341	0.7474	0.7433	0.7740	0.7980
**GF input**					
AUC ROC	0.9696	0.9678	0.9667	0.9763	0.9807
AUC precision-recall	0.7195	0.8028	0.7762	0.8269	0.8305

Shown in the table are AUC of ROC and precision-recall curves from CNNs with input size 100 × 100 × 12, 200 × 200 × 24, 400 × 400 × 48, multi-scale, and random forest.

**Table 3:** Effect of color image as input and random forest on false-positive reduction performance.

Sensitivity	0.95 (12 FN)	0.90 (23 FN)	0.85 (36 FN)	0.80 (48FN)	0.75 (60FN)
**Multi-scale CNN**					
GF	600	239	133	76	56
Color + GF	384	241	155	109	76
**Random forest with multi-scale CNN + hand-crafted features**					
GF	495	241	120	81	56
Color + GF	345	178	119	89	76

The number of FP candidates at different sensitivities in the range of 0.75–0.95 are compared.

Finally, sensitivity, specificity, precision, F1 score, and AUC of ROC from fourfold cross validation are shown in Table 4. The results were generated with all three strategies applied. Folds 1–4 represent the test mouse corresponding to cancer mice 1–4, respectively. In fold-2 with cancer mouse 2 as the test, we achieved a lower sensitivity because the GFP signal of metastases in the liver was dimmer compared to that of the other mice, and therefore the classification missed them. Having more cancer (+) mice for training will improve the CNN classification performance.

**Table 4:** Sensitivity, specificity, precision, F1 score, and AUC of ROC of fourfold cross validation.

	Sensitivity	Specificity	Precision	F1 score	AUC of ROC
Fold-1	0.8683	0.9812	0.5335	0.6604	0.9712
Fold-2	0.7440	0.9740	0.3563	0.4819	0.9466
Fold-3	0.9421	0.9761	0.4810	0.6369	0.9906
Fold-4	0.9038	0.9637	0.5482	0.6825	0.9754

**Figure 8:**
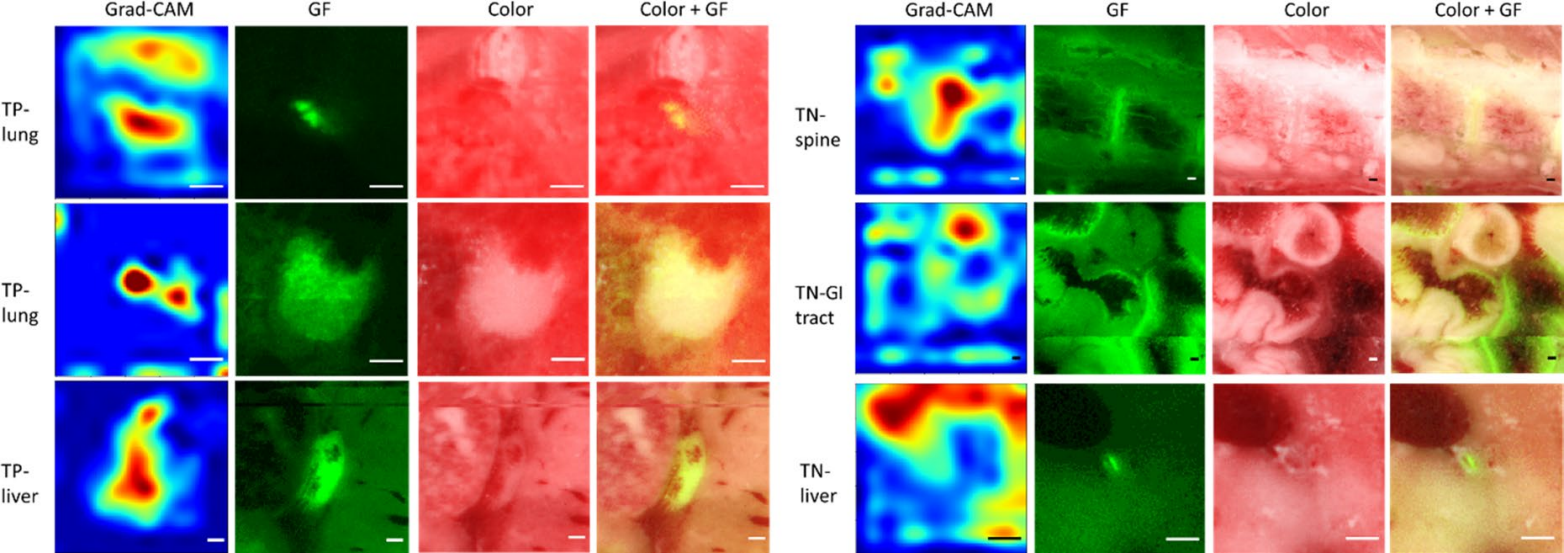
Grad-CAM for TP and TN candidates. The first two TP candidates are from the lung, and the last TP candidate is from the liver. The TN candidates are from the spine, GI tract, and liver. Scale bar is 200 µm.

The gradient-weighted class activation maps (grad-CAM)^37^ from three TP candidates and three TN candidates are shown in Fig. 8. The six candidates are shown in various input sizes, and the grad-CAM was generated with the corresponding CNN scale using color + GF image as the input. The first TP candidate is a micro-metastasis in the lung with a diameter < 100 µm. The grad-CAM shows that CNN utilizes information from the metastasis as well as the rib cage bone above the metastasis. However, the bone is not visible in the GF image. Therefore, the color image provides important anatomical information. The second metastasis is also from the lung and has diameter ~ 600 µm. The grad-CAM has strong response where the fluorescence intensity is high in the metastasis. Metastases bigger than 500 µm generally have a white appearance on the color image that could assist in classification. The TN candidate in the liver is auto-fluorescence from the bile duct in the liver. We can see that for the CNN, the vessel near the bile duct has the most weightage for making a cancer negative prediction. With grad-CAM, we revealed that our CNN is trained to focus on important fluorescence and anatomical information detected from color and GF images.

### Semi-automatic correction after classification

We developed a MATLAB GUI for easy and rapid semi-automatic exclusion of FP candidates and used Amira software for simultaneous inclusion of FN candidates. Human readers can scroll back and forth to identify FP candidates, and it is a simple, one-click process to select and exclude a FP candidate. As an example, before correction, cancer mouse4 exhibited FP candidates in the bone, spinal cord, distal colon, bile duct, spleen, kidney, and ear. Example FP candidates are shown in Fig. S1. Given the anatomical information from color images and proper training, an expert reader removed 78 FP candidates within 1 h, whereas the remaining 100 out of 178 FP candidates were missed annotations. The FN candidates included 3 candidates merged with the gall bladder and GI tract, 12 from highly necrotic adrenal glands, 3 in the brain, and 5 in the lung. To correct for FN candidates, the expert reader was instructed to focus on regions connected to the gall bladder and GI tract. FNs in highly necrotic adrenal glands were easy to detect, whereas FNs in the brain and lung required greater search time. In general, expert readers were able to include all FNs within 1 h.

### Tumor burden assessment

**Figure 9:**
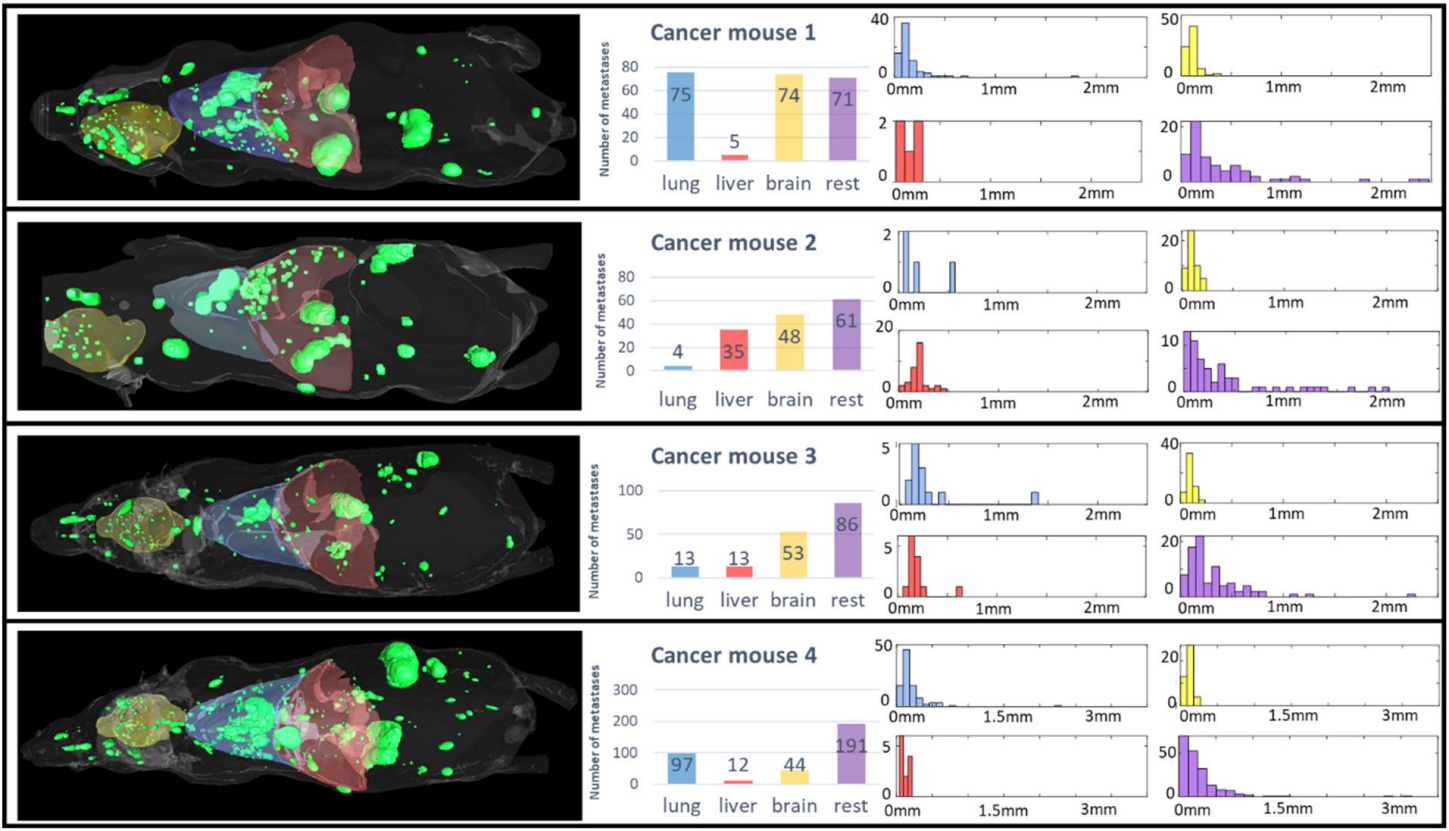
Assessment of tumor burden in four breast cancer mice. Three-dimensional volume rendering of the four cancer mice are shown on the left. Mouse body, brain, liver, lung, and metastases are shown in white, yellow, blue, red, and green, respectively. In the middle, the number of metastases in lung, liver, brain, and the rest of the body are shown. The right four subplots represent the histogram of radius of metastases in the lung, liver, brain, and the rest of the body with the same colors as the plot in the middle. There are no metastases bigger than 2.5 mm in cancer mouse 1–3, and there are 2 metastases in the range of 2.5–3 mm in mouse 4.

After segmentation and classification of candidates and semi-automatic correction of FP and FN candidates, we further quantified the size and distribution of metastases across the four cancer-bearing mice. Out of the 225, 148, 165, and 344 metastases from cancer mice 1–4, respectively, the anatomical distribution, number of metastases, and histograms of the metastasis radius in the lung, liver, brain, and rest of the body are shown in Fig. 9. The lung, liver, and brain masks were manually generated. In the brain, all metastases were micro-metastases with a radius < 0.5 mm. Most metastases in the liver were micro-metastases, except one metastasis found in cancer mouse 3. The lung mask included the heart region; therefore, metastases > 0.5 mm in cancer mice 2 and 3 were from the heart. However, there were metastases > 0.5 mm in the lung from cancer mice 1 and 4. In the rest of the mouse body, metastases were commonly found in the bone (e.g., spinal cord, mandible, spongy bone in femur and humerus), adrenal glands, and muscle. Metastases were also found in the pancreas, kidney, and ovary. Although metastases only grow in mice for 2–3 weeks, substantially large metastases > 2 mm formed in the adrenal gland, heart, and muscle tissue. The total tumor volumes in the four mice were 214.81 mm^3^, 199.89 mm^3^, 97.94 mm^3^, and 352.25 mm^3^.

### Evaluation of the method on KPC-GFP pancreatic tumor

**Figure 10:**
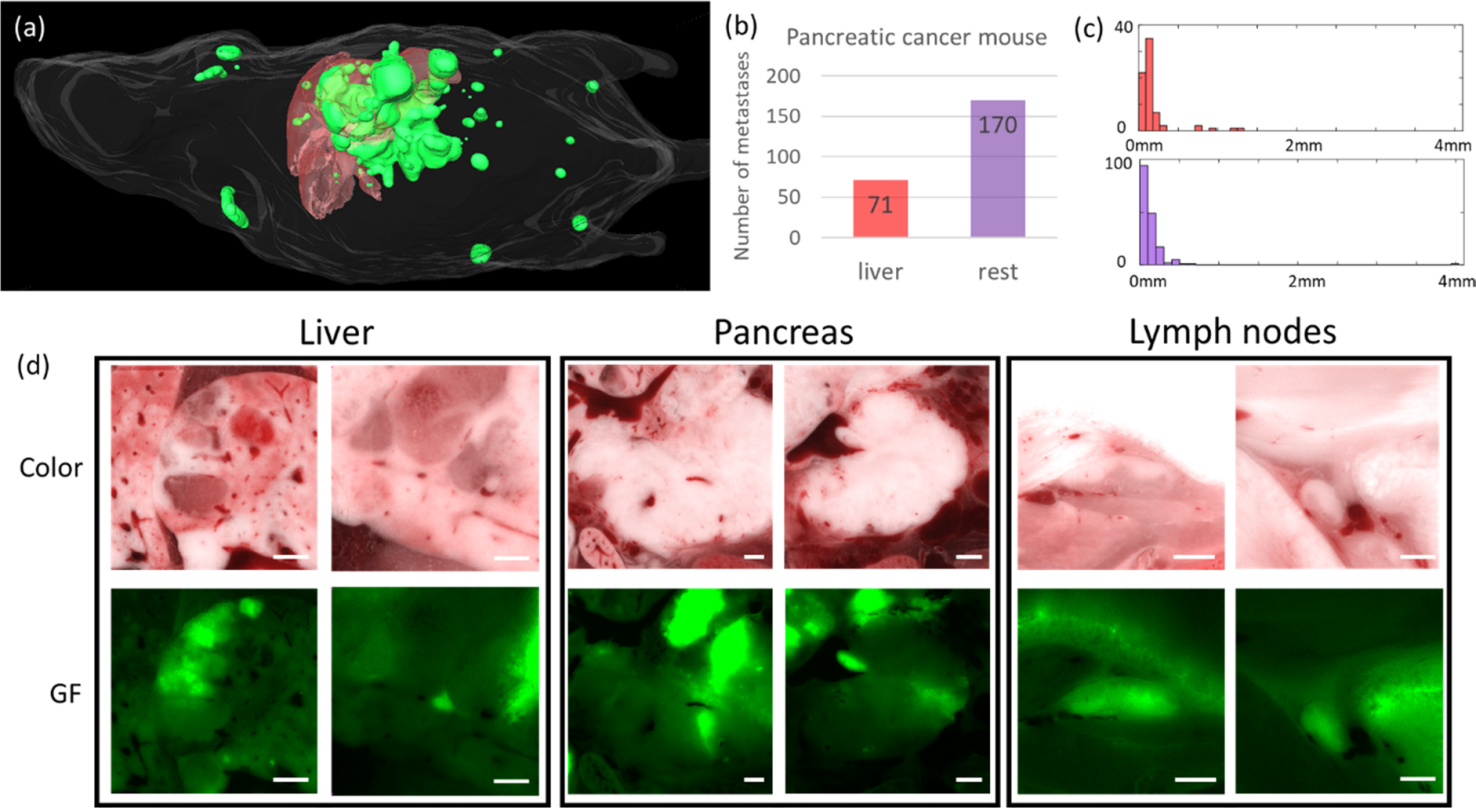
Assessment of tumor burden in a pancreatic cancer mouse. Three-dimensional volume rendering is shown in (**a**). Mouse body, liver, and metastases are shown in white, red, and green, respectively. The number of metastases in liver and the rest of the body are shown in (**b**). The two plots in (**c**) represent the histogram of radius of metastases in the liver, and the rest of the body with the same colors as the plot in (**b**). All metastases in the liver are smaller than 1.5 mm, and there is a large metastasis with radius 4.0 mm in the pancreas. Representative metastases in the liver, pancreas, and lymph nodes are shown in (**d**). For lymph nodes, the left one is the brachial and the right one is the inguinal. Scale bar is 100 µm.

With only minor modification and without additional deep learning, we tested the generalizability of our method on a mouse with KPC-GFP pancreatic cancer metastases. The workflow was the same as the segmentation of intra-cardiac breast cancer metastases. Since the tumor cell line was different, we adjusted some parameters easily determined from intuition. First, in the step “segment big-metastases candidates,” the GF image threshold was set to 60. Second, in the step “segment small-metastases candidates,” the threshold T_1_ was set to 8.0. We applied the trained deep learning model without modification. Deep learning was chosen rather than the random forest model with hand-crafted features because the latter was too finely tuned to the breast cancer model. The multi-scale deep learning model trained using the intra-cardiac mouse 1 and 2 were utilized to perform classification. We achieved sensitivity of 0.9286, specificity of 0.9236, precision of 0.2761, F1 score of 0.4257, and AUC of ROC of 0.9688, before any semi-automatic correction. In addition to common FP sites such as auto-fluorescent bones, bile ducts in the liver, gallbladder, alfalfa-free food remnants in the GI tract, lung airways, and ear, this mouse had FPs in the kidney and testis. FPs in the kidney were not related to tumor as we saw similar fluorescence signal in the negative control mouse with saline injected in the portal vein. FPs in the testis were likely obtained because all the breast cancer mice in the training data were female. We identified twelve FNs, suggesting a need to manually review results. Two were from large necrotic metastases in the pancreas, which had heterogeneous GF texture and irregular shape, different from the big metastases in the breast cancer model which had relatively homogeneous texture and shape similar to spheroid. Seven were from metastases neighboring the portal vein in pancreas, likely not chosen because they are similar to bile duct near vessels in the liver. The remaining three were from the brachial and inguinal lymph nodes, which are adjacent to the skin, giving tumor locations not present in the training data. After semi-automatic correction, the tumor burden is shown in Fig. 10. Example metastases developed in the liver, pancreas, and lymph nodes are shown in Fig. 10d. Approximating all the metastases to a sphere, the radius of the large pancreatic metastasis is 4.0 mm, whereas all metastases in the liver have radius < 1.5 mm. The total tumor volume is 299.80 mm^3^.

## Discussion

Our deep learning-based metastases segmentation software on cryo-images will make many preclinical research applications more efficient and robust, including imaging agents, imaging methods, cancer therapeutics, and tumor models. In this paper, we demonstrated metastases segmentation, quantification, and evaluation of the distribution in the intra-cardiac 4T1-GFP-Luc2 breast cancer mouse model, followed by a demonstration of generalizability to KPC-GFP pancreatic tumor mouse model. The 4T1 intra-cardiac model is an interesting experimental animal model for human mammary metastatic cancer, as it produces reliable bone metastases that are common in human cancer patients^38^. Such a model provides a good means for evaluating imaging agents^13^ and drug/biologic therapies^39^. However, metastatic cancer is particularly difficult to analyze, as tumors can be small and scattered over various types of tissue in cryo-images, thus making the human annotation time consuming and prone to error. The deep learning-based metastases segmentation software holds significant value by greatly reducing the time required for human intervention in the segmentation procedure from ~ 1 day to ~ 2 h. In addition, the human reader missed 23, 31, 22, and 94 metastases during the first-pass annotation, which corresponds to 0.8978, 0.7905, 0.8667, and 0.7267 sensitivity, respectively, in the four cancer mice. The segmentation software is more sensitive than humans at picking up micro-metastases.

We employed a segmentation procedure that is suitable for the current cryo-image intra-cardiac 4T1-GFP-Luc2 breast cancer mouse dataset. Although we did not compare the classification between 3D CNN and 2D CNN, 3D CNN provides more information and was deemed useful in previous lung nodule classification works^35,40^. Cancer (−) candidates might be visually similar to cancer (+) candidates in 2D and in a smaller bounding box view, such as the negative candidate in the bile duct (Fig. 5). Therefore, we implemented three strategies to assist false-positive exclusion: (1) multi-scale input and CNN, (2) color + GF image input, (3) random forest classifier with multi-scale CNN features + hand-crafted features. We next justify our strategies. First, multi-scale input and CNN capture information in the bounding boxes with different sizes and accommodate for various-sized metastases. Second, there is more anatomical information present in the color images than in the fluorescence images. Third, the random forest classifier directly utilizes multi-scale features and hand-crafted features for prediction; whereas multi-scale CNN fuses predictions from three scales instead of using multi-scale features for prediction. The segmentation process requires manual correction, which in turn could be used to further refine the model. The algorithm generalized fairly well to the pancreatic cancer model even though it contained tumors with different spatial distributions and visual characteristics than those in the breast cancer model. In the future, as we continue to use the software, we will include additional mice with corrected manual annotations to create a larger training dataset to improve the learning model.

Our software makes it possible to perform semi-automatic complete exclusion of auto-fluorescent candidates in cryo-images. Auto-fluorescence arises from lipofuscin, collagen, elastin, and red blood cells^41^, and is prevalent in tissues such as bone, bile duct, GI tract, lung airways, ear, spleen, and kidney. Although alfalfa-free food contains less chlorophyll than alfalfa mouse food, there are fluorescence signals that are comparable to the tissue auto-fluorescence level. Before classification, the number of auto-fluorescent candidates in the whole mouse was generally greater than 5000. After classification, most were excluded. With our semi-automatic correction tool, human readers that are trained to examine auto-fluorescence from healthy control mouse can quickly exclude FP candidates.

Our experiment demonstrated that both the intra-cardiac breast cancer and intrahepatic pancreatic tumor models give distributions of metastases that mimic their clinical patient counterparts. According to the cancer seed and soil theory, the organ micro-environment affects metastases growth. The most frequent target organs of metastasis are bone, brain, liver, and lungs^42^ and in our experiment, breast cancer metastases commonly formed in these tissues. Breast cancer brain metastasis is highly lethal in stage IV breast cancer patients. Selecting a suitable therapy for treating brain metastasis is still an unmet clinical need. Therefore, researchers are trying to develop mouse models of brain metastases. In our intra-cardiac model, a significant number of micro-metastases grows in the brain. The size of brain metastasis is small (< 0.25 mm), probably because of the reduced amount of cancer cells after blood–brain-barrier filtration and a less favorable tumor micro-environment. Big metastases grow in the lung, liver, bone, adrenal gland, and muscle with more seeds and cancer-favorable soil. Breast cancer invasion into tooth tissue is a rare finding^43^. However, of the four mice, we found metastases in the mandible and incisor teeth. We validated these metastases via auto-fluorescence from the mandible and incisor teeth of the control mouse. Clinically, pancreatic cancer commonly metastasizes to liver, celiac plexus, ligament of treitz, and lymph nodes, having a different 3D spatial distribution as compared to breast cancer metastases. Our analyses indicate that metastases in the intrahepatic mouse pancreatic tumor model tended to be found in these locations. CITAP makes it possible to study tumor models from different organ sites across many mice and draw statistically significant conclusions.

Further, we could calculate the cancer cell doubling time given the 3D volumes of metastases from cryo-imaging segmentations. Initially, 1 × 10^5^ cancer cells were injected in the breast cancer mice. After 2–3 weeks, metastases grew, and cryo-imaging was performed. With the assumption that 10% of each tumor were cancer cells and 90% were stroma and breast cancer cell diameter is 15 µm, there are 1.21 × 10^7^, 1.11 × 10^7^, 5.52 × 10^6^, and 1.99 × 10^7^ tumor cells in the four breast cancer mice, respectively. The average doubling times are 2.89, 2.94, 3.45, and 2.62 days, respectively, which are similar to the previously reported 2.2 ± 0.4 days^44^. This is much faster than human primary breast tumor with an average doubling time of 212 days^45^ and human breast cancer metastases in the lung with an average doubling time of 92 days^46^. We also calculated the doubling time of the biggest metastases in the four breast cancer mice from the adrenal gland, heart, and muscle, assuming that these metastases started off as a single cell. The doubling times are 0.92, 0.96, 0.93, and 0.88 days, which is much faster compared to that for the other metastases. Tumor dormancy^47^ reveals the capacity of circulating tumor cells, disseminated tumor cells, and/or micro-metastases to remain at low numbers after primary tumor resection. With genetic or epigenetic fluctuation or changes in the local micro-environment, dormant cancer cells could start aggressive colonizing. The intra-cardiac metastatic cancer model equipped with cryo-imaging elucidates metastases growth in different tissues. Further research on longitudinal studies to identify the tumor cell dormancy period and growth rate in different tissues can be performed, but it is beyond the scope of this paper.

In summary, we present cryo-imaging and metastases segmentation for the CITAP software platform. We demonstrate the segmentation algorithm and its unique application in imaging, quantifying, and evaluating metastases throughout the whole mouse body. Combined with our previous work^13,34^, the CITAP platform proves that it is uniquely suited for the evaluation and optimization of pipelines of technologies (imaging agents, imaging methods, therapeutics, tumor models, etc.) important for detecting, understanding, and treating metastatic cancer.

## Supplementary information

Supplementary Information.
