# Supplementary material for: Quantitative analysis of metastatic breast cancer in mice using deep learning on cryo-image data
Source: Sci Rep. 2021 Sep 1;11:17527. doi: 10.1038/s41598-021-96838-y (PMC8410829; doi:10.1038/s41598-021-96838-y)
Supplement: Supplementary file 1 — Supplementary Information. [file 41598_2021_96838_MOESM1_ESM.docx]

**Quantitative analysis of metastatic breast cancer in mice using deep learning on cryo-image data**

Yiqiao Liu^a^, Madhusudhana Gargesha^b^, Mohammed Qutaish^a^, Zhuxian Zhou^a^, Zhengrong Lu^a^, David L Wilson^a,b,c,*^

^a^Case Western Reserve University, Department of Biomedical Engineering, 10900 Euclid Avenue, Cleveland, Ohio, 44106, USA

^b^Bioinvision Inc, Suite E 781 Beta Drive, Cleveland, Ohio, 44143, USA

^c^Case Western Reserve University, Department of Radiology, 10900 Euclid Avenue, Cleveland, OH, 44106, USA

* dlw@case.edu

**Management of full resolution with chunks of data.** Due to the very large data sizes of cryo-imaging and the need for full resolution processing to analyze metastases, we need to process the input data in chunks. The size of chunks depends upon the available RAM and the total size of resident 3D arrays required for processing. Table 1 lists the intermediate arrays and specifies the data types, as well as provides results in an equivalent number of copies, where a copy is the size of an 8-bit array corresponding to a GF chunk. Processing requires eight copies to be present in RAM. The number of full resolution image slices in each chunk and the number of chunks are calculated at run time using equation (1). Each chunk consists of a fixed number of slices, except the last chunk, which contains the remaining slices. We assume that chunks overlap by 20% of the number of slices with chunks above and below. For the first and last chunk, there is overlap of 20% below and above, respectively. Further optimization is possible.

Table S1. Estimates of RAM memory allocation required for approximating the size of chunks. Simultaneously existing intermediate results in the RAM, their data type and size, and the equivalent number of copies of the original GF volume are shown.

| Intermediate results | Data type/size | Equivalent to #copies |
| --- | --- | --- |
| Original GF volume | 8-bit unsigned integer arrays/1 byte | 1 |
| Maximum LoG filtering response | Single-precision arrays/4 bytes | 4 |
| Seeds of candidates | 8-bit unsigned integer arrays/1 byte | 1 |
| Morphologically dilated seeds | 8-bit unsigned integer arrays/1 byte | 1 |
| Otsu segmentation result | 8-bit unsigned integer arrays/1 byte | 1 |

$$\#slices in a chunk= \frac{RAM size}{8\times{size}_{x}\times{size}_{y}}$$

$$\#chunks=ceil\left( \frac{\#slices in whole mouse-\#overlapping slices}{\#slices in one chunk- \#overlapping slices} \right)$$

$=ceil(\frac{\#slices in whole mouse-0.2 \times\#slices in one chunk}{0.8 \times\#slices in one chunk})$ (1)


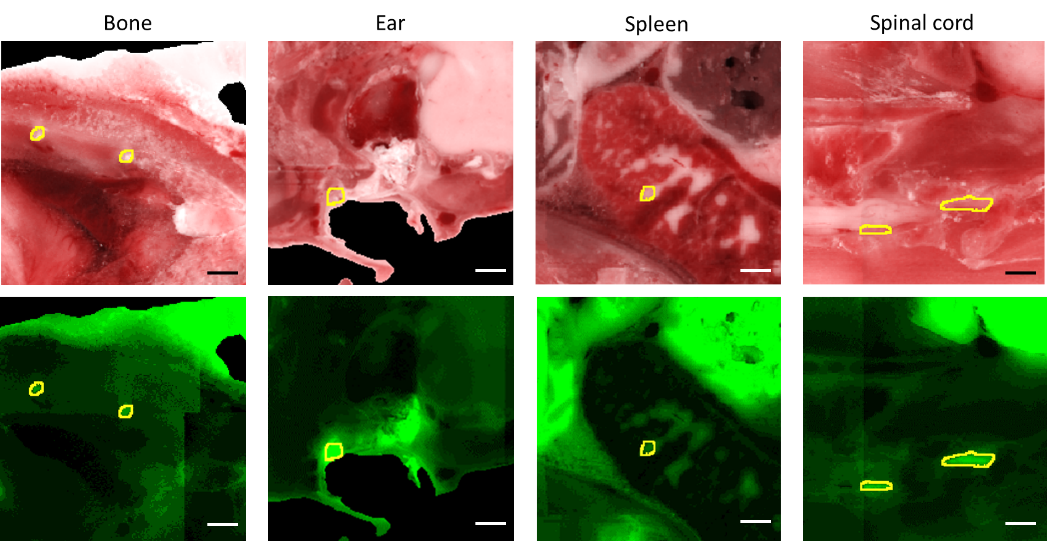


Fig. S1. Example FP candidates after classification in the bone, ear, spleen, and spinal cord. The candidate segmentations are shown in yellow contours in color and GF images. Given anatomical information, these FP candidates can be quickly removed with our Matlab GUI. Scale bar is 1 mm.
